# Draft Genome Sequences of Cloacibacterium normanense IMET F, a Microalgal Growth-Promoting Bacterium, and Aeromonas jandaei IMET J, a Microalgal Growth-Inhibiting Bacterium

**DOI:** 10.1128/genomeA.00503-18

**Published:** 2018-06-14

**Authors:** Shailendra Kumar Singh, Samuel R. Major, Haiyuan Cai, Feng Chen, Russell T. Hill, Yantao Li

**Affiliations:** a Institute of Marine and Environmental Technology, University of Maryland Center for Environmental Science and University of Maryland Baltimore County, Baltimore, Maryland, USA; b State Key Laboratory of Lake Science and Environment, Nanjing Institute of Geography and Limnology, Chinese Academy of Sciences, Nanjing, China

## Abstract

We report here the whole-genome sequencing results of two bacterial isolates, Aeromonas jandaei IMET J and Cloacibacterium normanense IMET F, that inhibit (possibly due to denitrifying gene clusters) and promote (possibly due to an ammonification system), respectively, the growth of the microalgal strains Scenedesmus HTB1 and Chlorella vulgaris 1807.

## GENOME ANNOUNCEMENT

Recent studies have shown that in bacterial-microalgal coculture, some bacteria may aid in recycling essential nutrients, such as nitrogen, sulfur, carbon, and phosphorus, and enhance microalgal growth (1–4). However, our understanding of the breadth, ecological significance, and chemical interaction of the microalgal-bacterial relationship is limited. Here, we present the whole-genome sequences of two bacterial strains, Aeromonas jandaei IMET J and Cloacibacterium normanense IMET F, isolated from chicken manure-enriched artificial algae ponds in Frederick, MD, USA. The effects of bacterial isolates on the growth of the oleaginous microalgal strains Scenedesmus HTB1 and Chlorella vulgaris 1807 were analyzed. A. jandaei IMET J inhibited the growth of both algal strains. In contrast, C. normanense IMET F has a “probiotic” effect, promoting algal growth in the bacterial-microalgal coculture.

To further understand the genomic foundation of these inhibitory and probiotic effects, the genomes of these two bacteria were sequenced. Genomic DNAs of both bacterial isolates were isolated using a Gentra Puregene Yeast/Bact. kit (Qiagen) for whole-genome analysis. The whole-genome libraries were prepared using the Illumina Nextera XT library preparation kit with 250-bp paired-end sequencing with an Illumina MiSeq instrument (Illumina, San Diego, CA, USA). Sequencing read quality was improved by adapter removal and trimming of low-quality bases using the Trimmomatic tool (5) on Galaxy (6). *De novo* assembly of all trimmed reads was performed with SPAdes version 3.11 (7), showing genome coverages of 276× and 217× for A. jandaei IMET J and C. normanense IMET F, respectively. The genome sizes were 4,726,205 and 2,842,441 bp for A. jandaei IMET J and C. normanense IMET F, respectively, whereas the G+C contents were 58.4% and 33.0%, respectively.

Gene annotations were performed with the NCBI Prokaryotic Genome Annotation Pipeline (PGAAP) version 4.2 and Rapid Annotations using Subsystems Technology (RAST) server (8). RAST annotation results show that A. jandaei IMET J, but not C. normanense IMET F, has denitrifying reductase gene clusters. These genes transform ammonia into N_2_O gas (9) and eventually reduce the nitrogen availability in media, which likely reduces the nutrient availability for algae growth. In contrast, C. normanense IMET F has two ammonification systems (whereas A. jandaei IMET J does not), Nap nitrate reductase (nitrate and nitrite) and Nrf nitrite reductase (nitrite to ammonium), at the periplasmic membrane of the algal cells (10). These systems may increase the availability of nitrogen in media, consequently promoting algal growth.

The genome sequences of A. jandaei IMET J and C. normanense IMET F provide a basis for understanding microalgal-bacterial interaction in a natural community ecosystem or industrial production ponds.

### Accession number(s).

The genome sequences have been deposited in DDBJ/ENA/GenBank under the accession numbers PRCV00000000 (Aeromonas jandaei IMET J) and PTPZ00000000 (Cloacibacterium normanense IMET F).
